# Understanding Inequities in Child Vaccination Rates among the Urban Poor: Evidence from Nairobi and Ouagadougou Health and Demographic Surveillance Systems

**DOI:** 10.1007/s11524-014-9908-1

**Published:** 2014-10-15

**Authors:** Abdramane Bassiahi Soura, Blessing Mberu, Patricia Elungata, Bruno Lankoande, Roch Millogo, Donatien Beguy, Yacouba Compaore

**Affiliations:** 1 University of Ouagadougou, Ouagadougou, Burkina Faso; 2 African Population and Health Research Center, Kenya, Nairobi

**Keywords:** Informal settlements, Slum, Child health, Vaccination, Ouagadougou, Nairobi

## Abstract

Studies on informal settlements in sub-Saharan Africa have questioned the health benefits of urban residence, but this should not suggest that informal settlements (within cities and across cities and/or countries) are homogeneous. They vary in terms of poverty, pollution, overcrowding, criminality, and social exclusion. Moreover, while some informal settlements completely lack public services, others have access to health facilities, sewers, running water, and electricity. There are few comparative studies that have looked at informal settlements across countries accounting for these contextual nuances. In this paper, we comparatively examine the differences in child vaccination rates between Nairobi and Ouagadougou’s informal settlements. We further investigate whether the identified differences are related to the differences in demographic and socioeconomic composition between the two settings. We use data from the Ouagadougou and Nairobi Urban Health and Demographic Surveillance Systems (HDSSs), which are the only two urban-based HDSSs in Africa. The results show that children in the slums of Nairobi are less vaccinated than children in the informal settlements in Ouagadougou. The difference in child vaccination rates between Nairobi and Ouagadougou informal settlements are not related to the differences in their demographic and socioeconomic composition but to the inequalities in access to immunization services.

## Background

Improving the health outcomes in the poorest countries of the world through increasing the number and quality of health services and creating public awareness on health issues in developing countries have dominated the political discourse of the World Health Organization (WHO) since the 1970s.^1^ While not neglecting the health of city dwellers, the focus, however, in terms of policies and programs in sub-Saharan Africa has long been geared toward reducing the gap between urban and rural areas. To date, policy makers still continue to emphasize on rural development efforts based of the perspective that socioeconomic and health conditions on average are better in urban than in rural areas.

Urban residents still exhibit lower mortality than rural ones in many sub-Saharan countries, but comparing averages can be misleading, and significant intraurban social and economic inequalities exist, which can result in substantial health inequities within cities.^2^^,^ 3 Indeed, during the last three decades, urbanization in sub-Saharan Africa took place in a context of deteriorating economies and poor planning and governance, resulting in unprecedented growth of slums. In sub-Saharan Africa in 2013, about 62 % of the urban population live in slums or slum-like conditions.^4^ Many poor and illiterate people live in these informal neighborhoods, which are characterized by unsanitary living conditions and the near absence of the public sector. In general, they exhibit high population densities, which are conducive for the spread of infectious diseases. Exposure to environmental hazards because of pollutants may also be high as these areas are often the unclean sections of cities.^5^ Urban health therefore has emerged as a major concern, particularly as it relates to urban slum residents and recent findings from sub-Saharan countries have increasingly challenged the urban advantage in health and other economic outcomes. Infectious diseases, child illnesses, and malnutrition indicators remain grim for urban slum dwellers, with substantial urban penalty for children in terms of higher mortality than the rest of the city and rural areas.^2^^,^ 6–10

While studies on informal settlements in sub-Saharan Africa have questioned the health benefits of urban residence, this should not suggest that informal settlements (within cities and across cities and/or countries) are homogeneous. Informal settlements in the region do not have the same level of basic social services. Majale evinced the diverse heterogeneity of low-income informal settlements in developing countries, which defied generalization, and in the particular case of Nairobi, illustrates the diversity of typologies of settlements that can be found within a single metropolitan area with variations across characteristics.^11^ While some informal settlements completely lack public services, others have access to health facilities, sewers, running water, and electricity, even if these services are inadequate.^12^ Moreover, poverty, pollution, criminality, and overcrowding as well as social exclusion and the types of diseases vary according to the settlement.^12^ There are few comparative studies that have looked at informal settlements across countries accounting for these contextual nuances. In this paper, we comparatively examine the health status of children living in Nairobi and Ouagadougou informal settlements where health and demographic surveillance systems have been set up, using immunization coverage as a proxy outcome. Ouagadougou, the capital of Burkina Faso, is a city of 1,475,839 inhabitants.^13^ Because of its rapid population growth, Ouagadougou experiences a rapid expansion of its urban space especially at the periphery, mostly in the form of informal settlements, where according to the 2006 census, about 22 % of its population live. In Kenya, according to the 1999 census, Nairobi, the capital city had a population of 2.1 million but the 2009 census showed that the population had grown to 3.1 million, an increase of 47.6 % in a decade. Estimates show that over 60 % of Nairobi’s residents live in slums or slum-like conditions (representing almost 2 million of the 3.1 million inhabitants of the city), characterized by limited access to water and sanitation, overcrowding and poor housing conditions, limited employment opportunities, insecurity of life and property, and marginal presence of the public sector.^10^^,^ 12 ^,^ 14 ^,^ 15 A corpus of studies have highlighted the particular significant disadvantages of the urban poor living in informal settlements, with respect to morbidity, access to health services, mortality, and risky sexual practices relative to other population subgroups, including rural residents.^16^^-^ 19

Building on the heterogeneity of informal settlements and evidence that settlement typologies can indeed be of operational relevance in policy articulation and improvement strategy formulation, more specifically settlement upgrading,^11^ we examine in this study the differences in child vaccination rates between Nairobi and Ouagadougou’s informal settlements. We further investigated whether the identified differences are related to the differences in demographic and socioeconomic composition between the two settings or rather to specific effects related to each of them (such as health policy differences).

## Data and Methods

### Study Site and Sample

We use data from the Ouagadougou and Nairobi Urban Health and Demographic Surveillance Systems (HDSSs), which are the only two urban-based HDSSs in Africa. Both are research and interventions platforms on population and health issues. The Ouagadougou HDSS was established in 2008 in five neighborhoods of the city.^20^ Two of these neighborhoods (Kilwin and Tanghin) are official districts, with full access to municipal services. The remaining three neighborhoods (Nonghin, Polesgo, and Nioko 2), whose data are used in this comparative study, are unplanned. These neighborhoods widely known as the “non loti” (literally “unloted” zones) are located at the urban periphery of the city, pushing the city’s boundaries further into the surrounding villages. They are governed by traditional land tenure systems and, until recently, were not served by municipal electricity and water services. Houses in these areas are mostly built from clay bricks. Households are usually small, made of single men or young nuclear families, who went there in search of affordable housing. These young families build houses in the hope that they will eventually own the land when the city’s authorities formally allocate the land. The population density is not necessarily high in these areas (42 inhabitants per hectare). With regard to social characteristics, people living in Ouagadougou informal areas are more often poorer, uneducated, and born in rural areas compared to people living in formal areas.^21^ In June 2013, the population covered by the Ouagadougou HDSS in the three informal settlements totaled 46,216 residents (defined as persons present in the area for at least 6 months).

The Nairobi Urban HDSS (NUHDSS) has been following residents of two slums in Nairobi city—Korogocho and Viwandani—since 2002. As of 2013, the NUHDSS has been following over 70,000 individuals, residing in over 25,000 households. Compared to the Ouagadougou informal settlements, those in Nairobi are more crowded (over 730 people per hectare). The two slums are located less than 10 km from the Central Business District (CBD) of Nairobi, Kenya’s capital city, and about 7 km from each other. They are characterized by a lack of basic infrastructure, high unemployment rates, poor water and environmental sanitation, poor housing, insecurity, violence, and poor health indicators.^10^^,^ 22 Consistent with the perspective on heterogeneity of settlements, the socioeconomic status and demographic composition of the two slums differ from each other. Located in the industrial area, Viwandani residents have relatively higher levels of education and employment as it attracts migrant workers to the surrounding industries. It therefore has higher socioeconomic status than Korogocho. Additionally, it consists of higher prevalence of single-person households. Korogocho on the other hand has a more stable population, with residents having generally lived there for a long period. Korogocho also has greater co-residence of spouses, and the family size is generally bigger.^23^ Residents of both Nairobi settlements are mainly rural–urban migrants and contrary to Ouagadougou’s informal settlements, they undergo rapid renewal of their population (about 21 % per year) due to high circular migration patterns.^24^

In the Ouagadougou HDSS, immunization data are updated every round while in the case of Nairobi, they originate from the Maternal and Child health follow-up study nested in the NUHDSS. Since September 2006, all children born to NUHDSS residents were recruited into this study, and vaccination details were collected in the first visit about 4 months after birth with follow-up visits repeated thereafter at 4-month intervals. The vaccination status of children is considered as at December 31, 2011 and analyzed for children 12 to 59 months who, according to the WHO time frame, should have received all the necessary vaccines before their first birthday.^25^ This age group was chosen in several studies to analyze the full immunization of children.^26^^–^ 29 The sample size is 3103 children in Ouagadougou and 1369 in Nairobi.

## Measures

### Outcome Variables

The paper focused on two outcome variables: incomplete vaccination and incomplete vaccination by the age of 12 months. Full immunization is understood in the sense of WHO recommendations that a child should receive BCG vaccine, measles vaccine, three doses of polio vaccine, and three doses of DPT (diphtheria, tetanus, and pertussis). Children who did not receive these vaccines are considered to have incomplete vaccination and coded “Yes” (1); those who received all the vaccines have complete vaccination and are coded “No” (0).

All the vaccines above should be administrated by the age of 12 months.^25^ It is possible that some children aged 12–59 months are fully immunized but did not receive all the vaccines before the age of 12 months. That is why in order to take into account the delay in vaccination time frame, we are interested in a second dependent variable which is full vaccination by the age of 12 months. Children who have not received all the vaccines before the age of 12 months are coded “Yes” (1) and those who received all vaccines are coded “No” (0).

### Independent Variables

The main independent variable is the place of residence (Nairobi or Ouagadougou). The other independent variables include two characteristics of the child: sex to examine whether there was discrimination against girls, and age to account for the effect of timing when the analysis focuses on full vaccination; and mother’s characteristics: age at child birth, length of residence in the slum community, religion, and educational level. A household level variable measuring the standard of living of the household is also controlled for in the analysis. The influence of these variables on child health has been well-documented in the literature.^30^

With regards to the standard of living, it is a complex phenomenon far from unanimous definition. In this study, we consider that the standard of living of a household reflects its ability to meet basic needs (food, housing, clothing …). Regarding its measurement, the scientific literature offers a variety of approaches, especially in terms of available data. They differ in general on the variables used to measure the standard of living as well as the method used to synthesize these variables in an indicator of standard of living. In particular, in social studies, several authors use information based on household’s goods and housing characteristics.^31^, ^32^ In the absence of information on household income, this latter approach was adopted in this work, and we created an index of standard of living using data on some households’ goods (TV, refrigerator) and means of transportation. The ownership of these goods can be markers of household living standards both in Ouagadougou and Nairobi. Two groups were defined: the less poor and the poorest.^1^

Proxies for standard of living based on household goods are not free from criticism. Indeed, from an analysis of five developing countries (Ghana, Guatemala, Jamaica, Pakistan, Peru, and Tanzania), Montgomery et al. showed that indicators of standard of living based on the household’s goods do not maintain a strong correlation with the standard of living as measured by consumption expenditure.^32^ However, they argue that because of the relatively high sample sizes in sociodemographic surveys, hypothesis tests based on such proxies are “*likely to be powerful enough to warrant consideration*” (p. 155).^32^ Moreover, Filmer and Pritchett showed the usefulness of asset index in establishing differences in long-run household wealth,^31^ and Gwatkin et al. concluded that asset ownership can be taken as a reasonable satisfactory proxy for consumption, in addition to being an indicator of economic status in its own right.^33^ Furthermore, it should be noted that in our comparative work, we are using a relative approach to poverty. Being poorer in Ouagadougou’s informal settlements may be better than being less poor in the Nairobi’s slums. Our goal is simply to see if controlling for the other characteristics, the fact of belonging to a socioeconomic group called “lower,” always has a negative effect on health.

### Analyses

The paper is based on an interpretation of proportions and odds ratios estimated in a pooled analysis where data from Ouagadougou and Nairobi were combined into the same dataset. This type of analysis (pooled data) is well known in epidemiology.^34^^,^ 35 In the social sciences or public health, it has been used by Hatt and Waters who combined Demographic and Health Survey (DHS) data as well as Living Standards Measurement Surveys (LSMS) data from several Latin American countries to study child morbidity (risk of diarrhea and risk of respiratory infection).^36^ It was also used by Rutstein who combined the DHS data from African countries to study the effect of birth interval on child mortality and child malnutrition.^37^ In general, the analysis of the pooled data from different surveys requires that standard errors be adjusted by using robust variance estimation methods such as bootstrap or Huber–White method.^36^ In this paper, we used bootstrap method with 1000 replications. There is no issue of weighting because all units that met a given criterion were included: resident children aged 12–59 months on December 31, 2011 in the Ouagadougou HDSS and children born from resident mothers and aged 12–59 months on December 31, 2011 in the Nairobi HDSS. This is similar to the analysis of a census data and it is not meaningful to consider weighting.

It is clear that such an analysis estimates a global effect for individual variables theoretically associated with child vaccination status. If a difference persists between Nairobi and Ouagadougou after these “average effects,” this difference is attributable to contextual specificities, such as access to health care, vaccination strategies, etc. These contextual specificities could even make that we cannot observe the same inequalities in child vaccination rates if the analysis was done separately for each city.

**FIGURE 1:**
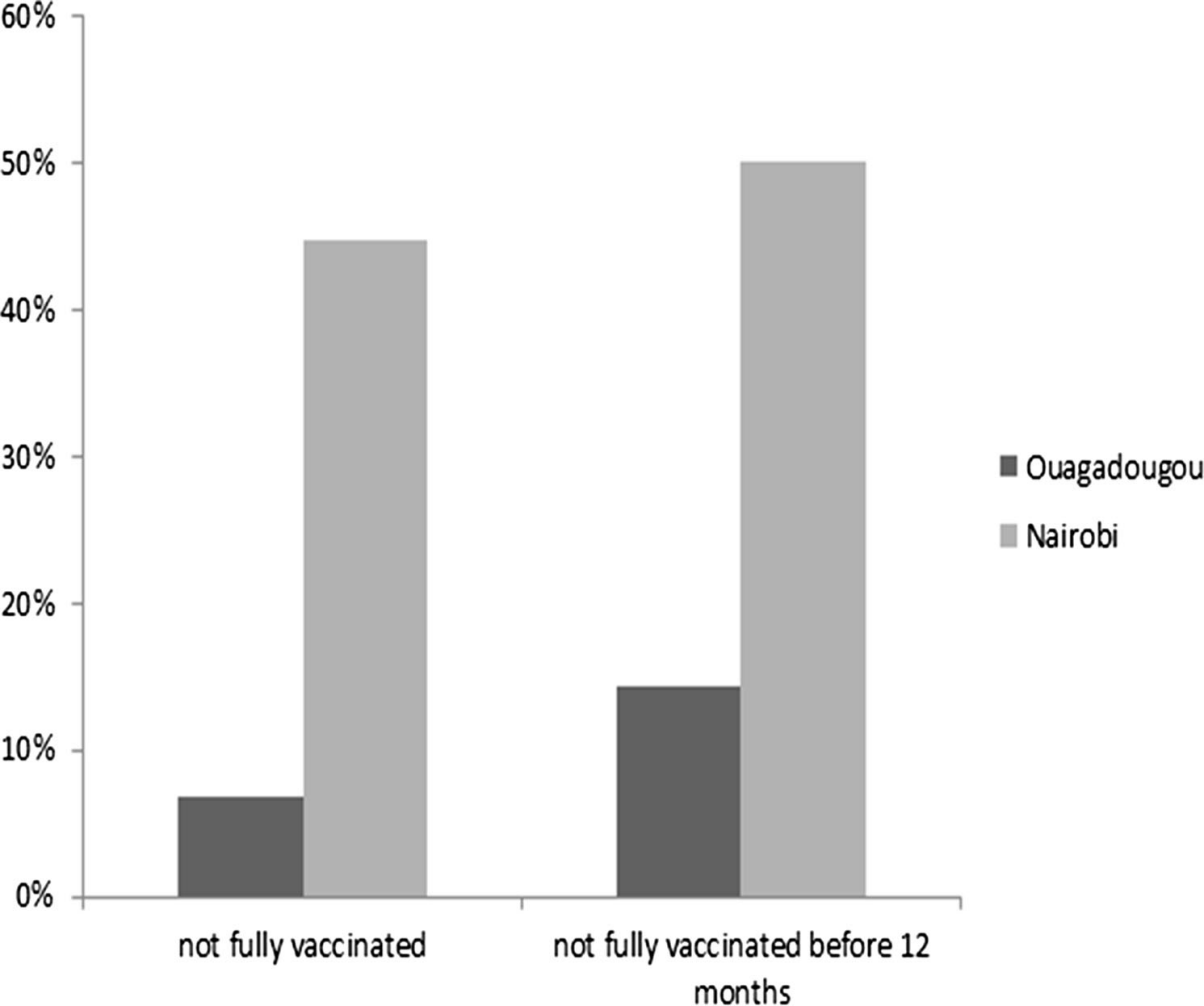
Percentage of children incompletely vaccinated by timeframe and place.

Descriptive statistics of the participants’ social and demographic characteristics were computed (Table 1). Bivariate analyses were conducted to examine sociodemographic differences in child vaccination (Tables 2 and 3; Fig. 1). We then run multivariate logistic regression to capture the influence of place of residence on child vaccination controlling for a number of other variables (Table 4). In our models, the explanatory variables are introduced in blocks to see how the difference between the two cities diminishes or enhances as the individual variables are taken into account. Four models were run. The first tests the basic effect of the place of residence (unadjusted effect, without controlling for any variable), and the second the mediating effect of demographic variables (sex of the child, length of residence of the mother in the slum/informal settlement, and age of the mother). The third model adds the effect of cultural factors (religion and educational level) and the last model tests the effect of the place of residence regardless of all the control variables. The age of the child is controlled for, when the analysis relates to incomplete vaccination without time frame.

**TABLE 1:** Distribution of children by individual characteristics in the two places

Variable	Sample for vaccination	
	Ouagadougou (%)	Nairobi (%)
Age of the child	***	
12–23 months	45.2	5.3
24–59 months	54.8	94.7
Sex of the child		
Male	49.7	48.6
Female	50.3	51.4
Mother’s length of stay in the slum/informal neighborhood	***	
Fewer than 5 years	64.3	55.9
5 years or greater	35.7	44.1
Age of mother at the child’s birth	***	
Under 20 years old	14.3	18.6
Between 20 and 34	79.8	75.9
35 years old and greater	5.9	5.5
Religion of the mother	***	
Christian	33.5	92.6
Other religion	66.5	7.4
Mother’s educational level	***	
None	62.3	2.1
Primary	24.2	69.0
Secondary and higher	13.5	28.9
Standard of living	***	
Poorer	48.5	29.3
Less poor	51.5	70.7
*N*	3103	1369

Significance at *p < 0.1; **p < 0.05; ***p < 0.001

**TABLE 2:** Percentage of children who have not received vaccines among those who were not fully vaccinated

	Ouagadougou	Nairobi
BCG	9.1	2.1
Polio0	9.1	18.5
DTP 1	15.3	2.7
Polio1	15.3	3.7
DTP 2	25.4	5.1
Polio2	25.4	5.9
DTP 3	43.1	11.4
Polio3	43.1	13.3
Measles	96.2	88.3
*N*	209	622

**TABLE 3:** Percentage of children incompletely vaccinated and odds ratios

Individual characteristics	Incomplete vaccination		Incomplete vaccination by 12 months	
	%	OR	%	OR
Age of the child				
12–23 months	7.67	1	–	–
24–59 months	23.9	3.79***	–	–
Place of residence				
Ouagadougou	6.7	1	14.4	1
Nairobi	45.4	11.53***	49.9	5.92***
Sex of child				
Male	18.4	1	25.1	1
Female	18.7	1.02	25.4	1.01
Length of stay in slum/informal neighborhood				
Fewer than 5 years	16.4	1	23.8	1
5 years or greater	22.1	1.44***	27.8	1.23**
Age of mother at child birth				
Under 20 years old	20.2	1	28.2	1
Between 20 and 34 years old	18.3	0.89	24.0	0.84*
35 years old and greater	17.7	0.85	22.7	0.75*
Religion of the mother				
Christian	27.1	1	32.6	1
Other religion	9.6	0.29***	17.5	0.44***
Mother’s education level				
None	8.7	1	17.6	1
Primary	28.4	4.16***	33.8	2.4***
Secondary and higher	21.9	2.93***	26.0	1.7***
Standard of living				
Poorer	16.0	1	23.5	1
Less poor	20.5	1.36***	26.6	1.18**
*N*	4472		4472	

Significance at *p < 0.1; **p < 0.05; ***p < 0.001

**TABLE 4:** Odds ratios associated with incomplete vaccination (logistic regression)

Variable	Incomplete vaccination				Incomplete vaccination before 12 months of age			
	M1	M2	M3	M4	M1	M2	M3	M4
Place of residence								
Ouagadougou	1	1	1	1	1	1	1	1
Nairobi	11.53***	10.45***	16.7***	17.08***	5.92***	5.84***	9.38***	9.54***
Age of the child								
12–23 months		1	1	1				
24–59 months		1.25*	1.25*	1.25*				
Sex of child								
Male		1	1	1		1	1	1
Female		0.99	0.99	0.99		1.00	1.00	1.00
Length of stay in slum/informal neighborhood								
Fewer than 5 years		1	1	1		1	1	1
5 years or greater		1.25**	1.20**	1.22**		1.10	1.06	1.07
Age of mother at child’s birth								
Under 20 years old		1	1	1		1	1	1
Between 20 and 34		1.03	1.07	1.07		0.93	0.96	0.96
35 years old and greater		0.96	0.95	0.93		0.81	0.78	0.78
Religion of the mother								
Christian			1	1			1	1
Other religion			1.27*	1.27*			1.22**	1.22**
Mother’s education level								
None			1	1			1	1
Primary			0.66**	0.67**			0.63***	0.64***
Secondary and higher			0.51***	0.53***			0.48***	0.49***
Standard of living								
Poorer				1				1
Less poor				0.86*				0.87*
Log Likelihood	−1708.84	−1703.58	−1692.27	−1690.94	−2228.14	−2226.14	−2205.76	−2202.20
Wald chi-square	745.61***	709***	689.20***	746.43***	548.01***	561.22***	639.91***	574.79***

Significance at *p < 0.1; **p < 0.05; ***p < 0.001

## Results

### Descriptive Analysis

Table 1 shows the distribution of children by individual characteristics. The difference between the two areas in terms of population distribution can be the root of their difference in terms of child vaccination rates. Regardless of the city, most children are aged 24–59 months and most parents fall into the middle age category (20–34 years). There is a relative balance between boys and girls. In both cities, the sample is mainly made of recent migrants in the slum community. Nairobi’s women are better educated than those of Ouagadougou. The two cities are different with regard to religion: Christianity is widespread in Nairobi while in Ouagadougou, other religions (mainly Islam) dominate.

Figure 1 shows that the vaccination coverage rate varies significantly by city. The immunization coverage of children is better in Ouagadougou than in Nairobi’s informal settlements. For instance, in Ouagadougou, 6.7 % of children between 12 and 59 months of age were not fully vaccinated while this proportion is more than 45 % in the case of Nairobi. In terms of vaccination by the age of 12 months, about half of the children in Nairobi’s informal settlements did not receive all the vaccines while this proportion is less than 15 % in Ouagadougou.

When a child has not received all the vaccines (209 cases in Ouagadougou and 622 in Nairobi), the vaccine against measles is the one missing most often (Table 2). In the case of Nairobi, other vaccines were missing in smaller levels (less than 15 % overall) while in Ouagadougou, in 25 % of cases, polio 2 and DTP2 were missing, and in 43 % of cases, polio 3 and DTP3 were missing (Table 2). Table 2 also suggests that the vaccine administration order is not well respected in Nairobi. In principle, BCG and Polio0 are given at birth. Polio1 and DTP1 are administered together, Polio2 together with DTP2, and DTP3 and Polio3 together. According to WHO, Polio1 and DTP1 should be given at 6 weeks. The two other subsequent doses should follow at intervals of 4–8 weeks each.^25^ The vaccine against measles is the last one to be given (between 9 and 12 months of age). This simultaneous administration of vaccines does not appear to be met in Nairobi since the proportions are not the same for vaccines that should be administered together (Table 2). In addition, 18.5 % of children who were not fully immunized were not vaccinated against polio at birth while this proportion is lower for Polio1 (Table 2).

The unadjusted odds ratios (Table 3) show that children in Nairobi’s slums are 11.5 times more likely to be incompletely vaccinated than children in Ouagadougou’s informal settlements. This risk is estimated to be 5.9 times higher for children in Nairobi, when it comes to being fully vaccinated before the age of 12 months.

We find that several control variables are also significant. This is the case in relation to the mother’s educational level and the standard of living (Table 3). However, these two variables maintain a counterintuitive relationship with child vaccination. For example, the proportion of children who have not received all the vaccines increased from 8.7 % when the mother is uneducated to 21.9 % when the mother completed high school. Among the mothers with primary education, this proportion is estimated at 28.4 %. The relationship with the standard of living also shows that incomplete vaccination rate is higher among the least poor families (Table 3). This unexpected result could be due to confounding factors which are not yet taken into account. At the bivariate level, the results also highlight an advantage for the children of non-Christian mothers. They are better vaccinated (Table 3).

The length of residence of the mother in the slum community also has a significant relationship in an unexpected direction. Children of recent migrants in the slum community (<5 years) have a lower risk of incomplete vaccination (Table 3). The child-specific characteristics, i.e., gender and age, show a significantly higher risk of incomplete vaccination among children aged 24–59 months compared to those who were less than 24 months old.

### Multivariate Analyses

The significant relationship which was observed in the bivariate analysis between the place of residence and each of the two dependent variables (incomplete vaccination and incomplete vaccination before 12 months of age) is clearly noticeable in Table 4 (models 1). In general, the gradual inclusion of control variables did not diminish the effect of the place of residence (Table 4). All else being equal, the disadvantage of the Nairobi slums’ children is real (Table 4, models 4). Compared to children in Ouagadougou’s informal settlements, those in Nairobi have 17.1 times greater risk of not receiving all vaccines. This risk is estimated to be 9.5 in the case of incomplete vaccination before 12 months.

Regarding the control variables, the counterintuitive relationship that was observed in the bivariate analysis between education and child vaccination rate, as well as between wealth index and child vaccination rate, disappeared (Table 4, models 4). These relationships are in the expected direction indicating a lower risk of incomplete vaccination among children whose mothers are more educated and in the less poor families (vaccination before 12 months of age specifically). Adjusted relationships between mother’s religion and child vaccination rate are also consistent with our intuitive expectations (Table 4, models 4). The risk of incomplete vaccination is higher among children of non-Christian mothers. Table 4 also shows all things being equal, children whose mothers stayed longer in the slum community (more than 5 years) are 22 % more likely to be incompletely vaccinated compared to those of recent migrants (Table 4, model 4). Estimates show that this ratio is no longer significant in the case of incomplete vaccination before the age of 12 months.

## Discussion

Multivariate results showed that immunization coverage is lower among children in the slums of Nairobi than among their counterparts living in informal settlements in Ouagadougou. This difference in child vaccination coverage may be a result of difference in immunization policies and/or their implementation. Burkina Faso has adopted the Extended Program on Immunization since 1980 and designed its implementation with the goal of reaching more vulnerable people.^38^ Thus, mass vaccination campaigns complement routine immunization given in clinics. Regarding polio vaccine, campaigns are often organized as part of the global initiative to eradicate polio, and during which the advanced strategy called “door to door” is used. In this strategy, vaccinators roam neighborhoods, from house to house, looking for targeted children. Unlike polio, vaccines administered by syringe require nurses, who unfortunately are not numerous, making it difficult to implement the “door to door” strategy. In this case, the vaccination is performed at the health center or outside the health center, in a suitable public place. Whatever the strategy used, media and community workers are mobilized to inform the people and explain the benefit of vaccination for children. The implementation of this mobile vaccination strategy in Burkina Faso gives pride of place to geographic targeting.^39^ The idea is that the poorer an area, the more it is at risk and therefore requires more attention.^40^ An example is the great emphasis put on rural areas during national immunization campaigns.^41^ The Ouagadougou informal settlements, which were villages engulfed by the urban sprawl, also benefit from this effort.

Vaccination strategies in Kenya are more or less the same as in Burkina Faso (routine immunization in clinics, door-to-door strategy, and static centers in strategic sites). But mobile strategies (more frequent in Ouagadougou informal settlements) are rare in the slums of Nairobi. In the latter, there are some community-based clinics providing healthcare services including immunization. One example of those clinics is Alice Health Services in Mukuru Kwa Njenga in Nairobi, a slum area of more than 100,000 inhabitants.^42^ This clinic receives vaccines free of charge from Kenyan government and sends a monthly report (through the Division of Vaccines and Immunization) on the number and types of vaccines administered. Every Sunday, the clinic organized a campaign to vaccinate children against tuberculosis, polio, measles, and pneumonia. Although the aim of the clinic is to improve access of the poor to health services, these services are not free and in the context of extreme poverty, the reported 30 shillings (about 0.35 USD) vaccination fee may discourage some parents from vaccinating their children properly.^2^ It is true that two community health workers are recruited to educate reluctant parents, but this number may not be adequate for a population of over 100,000 people.

In summary, the differences in child vaccination rates between Nairobi and Ouagadougou informal settlements are, in our view, a result of inequalities in access to immunization services. It is also possible that Nairobi slums residents are more exposed to adverse health behaviors than the residents of Ouagadougou informal settlements. Indeed, although families in the slums of Nairobi have an advantage in terms of possessions, compared to those in Ouagadougou informal settlements, their daily life may be generally more distressing, with people not having enough resources to meet all their basics needs (paying for rent, water, food, health, etc.). Concerns for the day-to-day life can lead a parent to pay less attention to his/her child’s health, especially when it comes to preventing a disease whose symptoms are not yet visible. Consequently, children living in slums have been shown to be less likely to be vaccinated and have higher infant morbidity and mortality rates.

With regard to inequalities revealed through control variables, it appears that the disadvantage of children increases with the length of residence in the slums. The longer the duration of residence, the greater the child runs the risk of being incompletely vaccinated. This result reinforces the idea that the daily difficulties which mark the lives of slum dwellers can lead them to pay little attention to their health or the health of their children. It is possible that people arrive in the slums with a health behavior inherited, probably better than that prevailing in the slum community. For instance, Konseiga found that children of migrant households in Nairobi slums experience 39 % higher morbidity compared to children left behind in the rural country homes.^43^ The importance of the mother’s educational level in child vaccination status was present in all the models. This relationship is the most commonly observed in child health studies, due to the strong behavioral change induced by education. A health benefit among children of Christian mothers was also observed, due to the fact that in sub-Saharan Africa, Christians seem more open to allopathic medicine 44 and more positively disposed and less likely to oppose primary health care utilization around vaccination.

This comparative study has some limitations. First, the cross-sectional nature of the data used requires caution in interpreting the results, especially in making causal inferences. Then, there is a potential selection bias in the sample. It is possible that children less vaccinated have died and those who survived are those who were best vaccinated. Another selection bias relates to the Maternal and Child Health Study. Since this survey covered children born under surveillance, those who arrived by migration and were less than 5 years old were not taken into consideration. It is important to note, however, that there is small number of parents who usually migrate to the Nairobi’s slums with their children, and most children were born in the slums. Another limitation is that we could not control for potential confounding variables, as they were not collected during the surveys (e.g., parent’s knowledge and attitudes towards vaccination, place of delivery, and antenatal care). Finally, another limitation is related to the variable capturing standard of living. We classified households in informal settlements in Nairobi and Ouagadougou on the basis of goods and means of transportation. These goods do not have the same values from one place to another. However, we used a relative poverty approach to test if belonging to a “lower” socioeconomic group is always associated with a disadvantaged health status.

Building against the backdrop of the foregoing, our results highlight the importance of addressing the structural socioeconomic hindrances to full and timely immunization in Nairobi. Our results also highlight the disadvantage of long-term migrants in the slum community, less educated mothers, and women of other religious persuasion beyond Christianity in access to child immunization, calling for targeted context sensitive interventions.

## Footnotes

1 The poorest household travels on foot or bicycle and does not have a TV or refrigerator. The less poor household owns a motorbike or a car. They may have a refrigerator or a TV.

2 The 30 shillings (0.35 USD) vaccination fee corresponds to the price of about 1/3 kg of rice in the local market. Using a household survey conducted in 2004, the World Bank has estimated that 50 % of the Nairobi slums’ households earn less than 40 USD per capita per month.^35^
